# Towards a molecular basis of ubiquitin signaling: A dual-scale simulation study of ubiquitin dimers

**DOI:** 10.1371/journal.pcbi.1006589

**Published:** 2018-11-16

**Authors:** Andrej Berg, Oleksandra Kukharenko, Martin Scheffner, Christine Peter

**Affiliations:** 1 Department of Chemistry, University of Konstanz, Konstanz, Germany; 2 Department of Biology, University of Konstanz, Konstanz, Germany; Bogazici University, TURKEY

## Abstract

Covalent modification of proteins by ubiquitin or ubiquitin chains is one of the most prevalent post-translational modifications in eukaryotes. Different types of ubiquitin chains are assumed to selectively signal respectively modified proteins for different fates. In support of this hypothesis, structural studies have shown that the eight possible ubiquitin dimers adopt different conformations. However, at least in some cases, these structures cannot sufficiently explain the molecular basis of the selective signaling mechanisms. This indicates that the available structures represent only a few distinct conformations within the entire conformational space adopted by a ubiquitin dimer. Here, molecular simulations on different levels of resolution can complement the structural information. We have combined exhaustive coarse grained and atomistic simulations of all eight possible ubiquitin dimers with a suitable dimensionality reduction technique and a new method to characterize protein-protein interfaces and the conformational landscape of protein conjugates. We found that ubiquitin dimers exhibit characteristic linkage type-dependent properties in solution, such as interface stability and the character of contacts between the subunits, which can be directly correlated with experimentally observed linkage-specific properties.

## Author summary

Post-translational modification of proteins by covalent attachment of ubiquitin is a key cellular process, regulating for example the fate and recycling of proteins. We present a new method to combine multiscale simulation with advanced analysis methods to characterize the states of ubiquitin-ubiquitin conjugates. We found that the linkage position affects the conformational space of ubiquitin dimers, determining the number and stability of relevant states, the character of subunit contacts and the nature of the surface exposed to possible binding partners.

## Introduction

Ubiquitylation is a selective process mediated by a complex enzymatic cascade and involved in the regulation of many cellular processes [1]. Usually, ubiquitin (Ub) is covalently attached to substrate proteins via isopeptide bond formation between its C-terminal carboxylate group and the *ϵ*-amino group of a substrate’s lysine residue. Since Ub itself contains seven lysine residues and each of these as well as the N-terminal *α*-amino group can be ubiquitylated, substrate proteins can either be mono-ubiquitylated or modified by an in principle sheer unlimited number of different types of Ub polymers (Ub chains) [2]. Homotypic Ub chains, i.e. within one chain Ub moieties are linked via the same lysine residue or via the N-terminal methionine, are the best understood chain types with respect to structure and function [3]. For example, in a simplified view, K48-linked Ub chains target proteins to the 26S proteasome for degradation, while K63-linked chains signal modified proteins for non-proteolytic fates. The “Ub code”, i.e. the relation between the linkage type and the fate of the modified protein, is presumably mediated by different conformations of differently linked Ub chains [4]. The latter are in turn recognized by proteins harboring Ub binding domains (UBDs) that show either relative or absolute selectivity for different linkage types and determine the eventual cellular signal [5].

Due to their functional and physiological relevance, Ub chains and, in particular, Ub dimers have been a popular object for structural analysis by X-ray crystallography [6–9] and NMR spectroscopy [10–16]. The data clearly indicate that Ub dimers adopt different stable conformations that vary in their extent of inter-domain contacts. However, the structures available represent a subset of the entire conformation space that can be occupied by individual Ub dimers. The hydrophobic patch, as an example, that was reported to serve as an interaction hot spot for K48-linked chains, is apparently not accessible in various structures that were determined for this linkage type [17]. Consequently, additional efforts are required to elucidate the entire conformational ensemble of Ub dimers and, thus, the Ub code [18].

**Fig 1:**
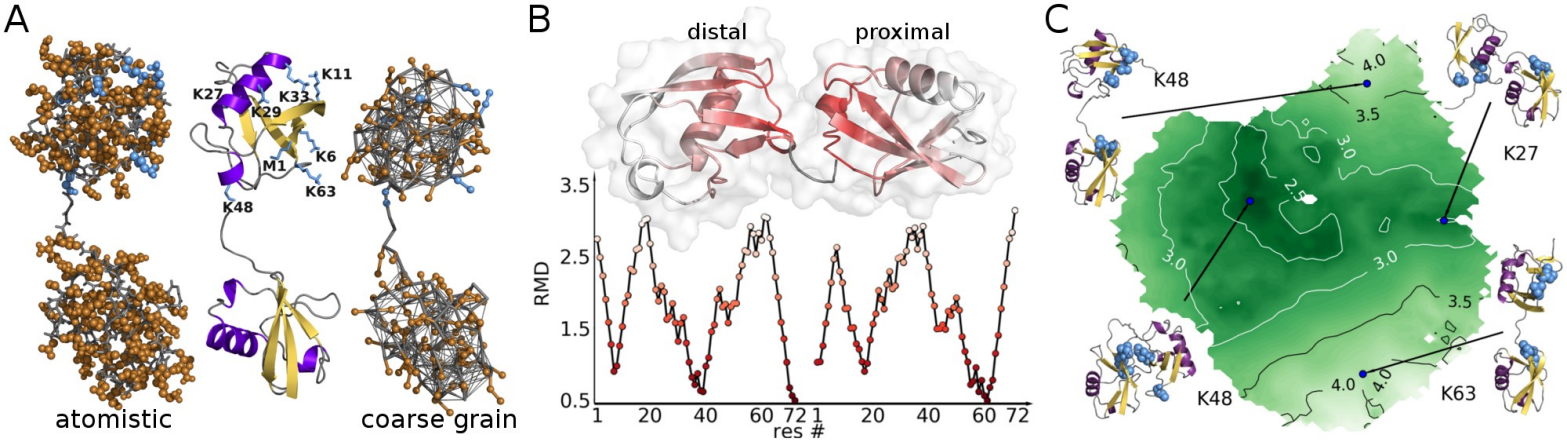
Ubiquitin dimers. (A) Proximal subunit linked via K48 to C terminus of distal subunit. Left: atomistic representation (gray: backbone atoms, brown: side-chains atoms, blue: lysine residues, i.e. alternative linkage positions, on proximal chain). Middle: cartoon representation of secondary structure. Right: coarse grained (CG) representation including supportive elastic network. (B) Residue-wise minimum distance (RMD in nm) for a K48-linked diUb structure. Residues are colored according to RMD (nm) values shown in diagram. (C) Sketch-map projection of all CG simulation structures (all linkage types). Coloring according to center of geometry distance between Ub subunits. Labels according to the linkage type illustrate conformational characteristics of certain map regions.

Molecular dynamics (MD) simulation is ideally suited to complement experimental data and to provide novel insights into properties of Ub dimers, like the nature and thermodynamic stability of distinct conformations in solution. Although Ub was in the focus of several computational studies, the full conformational space of Ub dimers has not been described by MD simulations so far [19–21]. Due to the computational cost of atomistic sampling, the equilibrium between different conformations is hardly accessible by standard atomistic MD techniques for a system of that size. A common method to overcome time and size limitations of atomistic MD simulations is coarse graining (CG) [22–25]. By uniting several atoms into one bead, the number of degrees of freedom can be drastically reduced (Fig 1A). Additional speedup is gained from softer potentials which allow larger time-steps and faster effective kinetics. On the downside, reduction of resolution inevitably limits the capability of a CG model to correctly reproduce all properties of a system. Therefore, in the present study, we pursued a dual-scale approach that takes advantage of CG and atomistic levels of resolution to simulate all 8 natively linked Ub dimers [26, 27]. Thus, we managed to sample the conformational phase space of each dimer on the timescale of 120 *μ*s. We introduce a new method to characterize and compare conformational free-energy landscapes of protein conjugates. This enabled us to systematically connect simulations on different resolution levels and to provide a quantitative measure for the similarity of differently linked Ub dimers (diUbs). We obtained a reliable atomistic description of their respective conformational characteristic which is in good accordance to known experimental data and can serve as an explanation for linkage-specific biological function.

## Results

### Characterization of diUb structures

Key to the identification of conformational states is finding suitable collective variables (CVs) that capture the characteristic features of a system. Since the number of CVs is often very large, dimensionality reduction techniques are applied that allow the data to be projected into a two or three dimensional representation for visualization and further interpretation [28]. For the characterization and comparison of diUbs, we identified a high-dimensional (144D) set of CVs that describe the multi-domain structure by internal coordinates between the two Ub moieties and projected these data to a 2D representation to obtain estimates of the free-energy landscape.

#### Collective variables

To qualify as suitable descriptors in this study, CVs had to meet the following requirements: They (1) are capable of describing structures from both atomistic and CG simulations, i.e. rely on coordinates which are present in both models, (2) contain information about the relative positioning of the two subunits in diUb, and (3) allow a comparison of differently-linked diUbs. We defined Residue-wise Minimum Distances (RMDs) which fulfill these criteria: A given diUb structure (consisting of two Ub moieties of 72 amino acid residues each; the highly flexible residues 73-76 of Ub were not considered) is described by a vector of 144 minimum distances between the two subunits. More specifically, for each of the 72 C_*α*_ atoms in the distal moiety the minimum distance to the C_*α*_ atoms in the proximal moiety is calculated and vice versa (see Fig 1B and S1 Text for a more detailed explanation). Note that focusing on the C_*α*_ atoms, which correspond to backbone beads in the CG model, allows a seamless linking between the CG and atomistic levels of representation. We found that this RMD vector well embodies the relevant information about the distance and the relative orientation between the domains and serves as an ideal basis for dimensionality reduction and conformational clustering.

#### Sketch-map

To obtain comprehensible low dimensional representations of the simulated data, we used a multi-dimensional scaling (MDS) approach called sketch-map [28–30]. This method is very well suited to project a highly nonlinear conformational space by iteratively minimizing a nonlinear fit function and by focusing on the intermediate range of distances between data points. Importantly, sketch-map can be very efficiently used for very large sets of simulated data: the main minimization procedure is applied to a subset of representative data points (landmarks). All other points are projected based on their relative positions to those landmarks.

To illustrate the effect of using sketch map on the RMD CVs of diUb, CG simulations of all linkage types were projected into one graph (for further details on sketch-map algorithm and projection see S1 Text). The resulting sketch-map (Fig 1C) has a circular shape and—quite exceptionally for a nonlinear MDS procedure—allows a physical interpretation of the data-point positions and an assignment of structural characteristics to certain regions: compact structures (with a low center of geometry distance between the domains) are found in the center of the map, while open structures lie in the outer region. Structures, where the two domains are interacting via the *β*-sheets of the Ub monomers, appear on the left side, while those interacting via the *α*-helices are on the right. Thus, we have obtained a physically interpretable landscape of diUb conformations based on which we can now not only cluster simulated structures but even compare and classify the behavior of differently linked chains.

### Conformational sampling

#### Coarse grained simulations

CG simulations were performed with a modified MARTINI force field. They were started from two different open initial conformations for each diUb linkage type (S2 Fig), which had been constructed by covalently linking two Ub monomers (PDB-ID: 1UBQ). From each of those 16 initial conformations, six independent runs with different initial velocities were performed for 10 *μ*s, resulting in a total CG simulation time of 960 *μ*s (not accounting for the additional speed-up of CG kinetics by a factor of 4-8 compared to atomistic simulations [24]). In contrast to comparable atomistic ones, the CG simulations were able to visit repeatedly different conformations and escape local free-energy minima (S3 Fig). Thus, despite the fact that even this extensive CG sampling is not yet fully converged, the Boltzmann inverted probability density (−*kT* ln(*p*)) in the 2D sketch map projection described above can serve as a rough estimate for the free energy landscape of diUb conformations.

**Fig 2:**
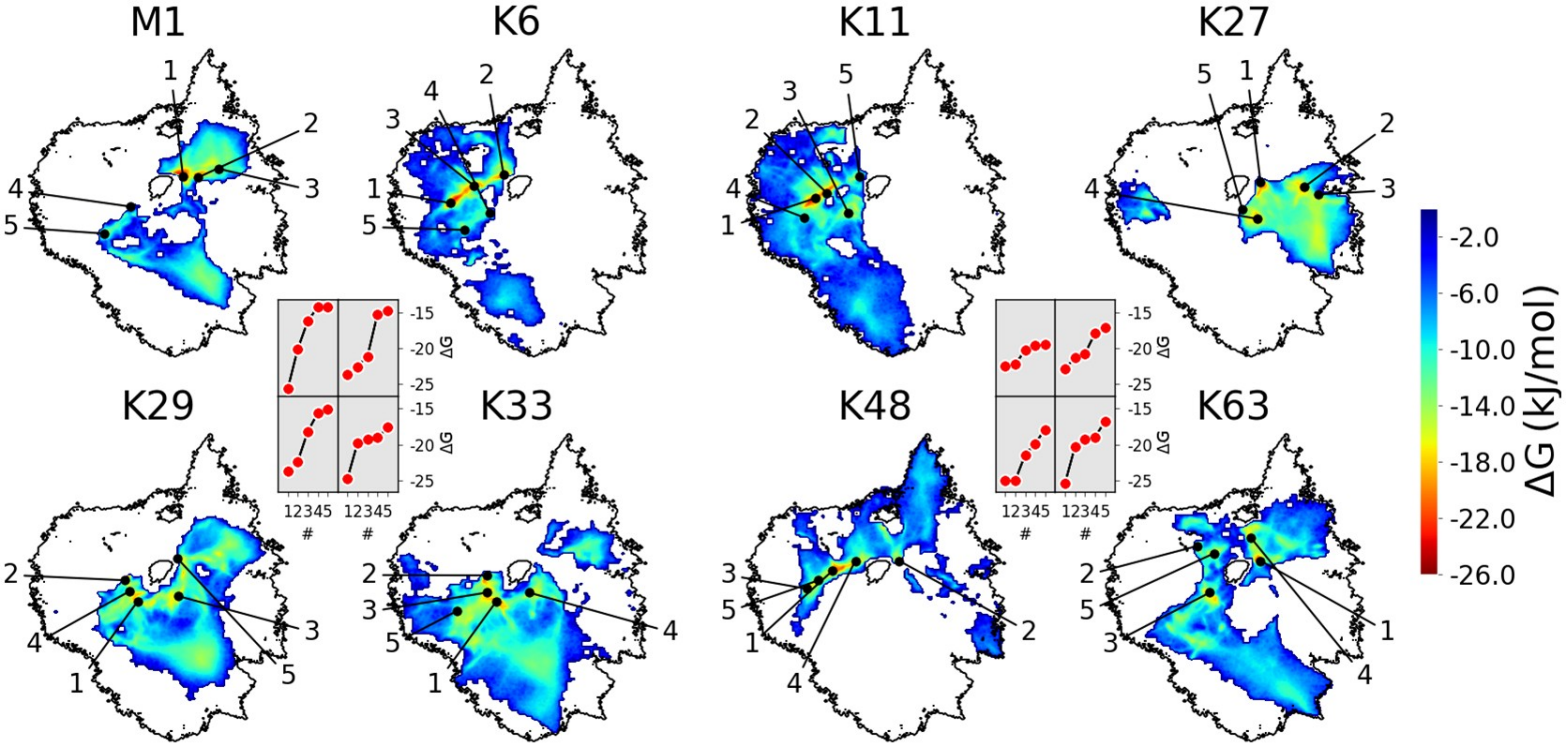
Conformational landscapes of diUb. Sketch-map projected RMD data. Black circular shapes: rim of combined landscape of all linkage types (Fig 1C). Colored maps: Boltzmann-inverted probability distributions from CG simulations of individual linkage types. Black points: five lowest free-energy minima (respective energies shown in insets).

Fig 2 shows the space visited by the differently linked diUbs. All linkage types exhibit several local minima in the conformational free-energy landscape in areas denoting compact structures (black points in Fig 2, insets show the values of the depths of the indicated minima). The number, spatial extension and relative depth of the basins in the conformational landscape can be used for a qualitative comparison with experimental ensemble data. These had shown that most diUbs indeed constitute a highly dynamic ensemble of structures with transient non-covalent contacts between the Ub subunits—with the notable exception of K48- and K6-linked diUb [15]. This is in good qualitative agreement with the behavior found in the simulations, as will be analysed in more detail below. A qualitative visual inspection of the visited areas of the landscape yields—not unexpectedly—large similarities for linkage types that are located in close proximity on the Ub surface, such as K6 and K11, K29 and K33, or K63 and M1. Notably, K27-linked diUb stands out. In particular, it differs significantly from K29- and K33-linked diUb—in spite of the sequence proximity—which is in good agreement with experimental observations [16, 31]. A more quantitative comparison of the linkages is presented below.

#### Atomistic simulations

Atomistic simulations were performed with two types of initial structures: (1) twelve simulations per linkage type were started from the same open conformations as the CG simulations (but with a runtime of 50 ns each) which results in a total simulation time of 4.8 *μ*s; (2) from CG conformations that were backmapped to the atomistic level following ref. [32] (the backmapped structures are shown in S4 Fig). In the latter case, structures were extracted from the four lowest free energy minima of the CG landscapes of each linkage type and simulated for 10 ns each, additionally 10 structures were selected randomly around each of those minima (simulated for 3 ns each) which sums up to ∼1.3 *μ*s simulation time for the backmapped structures. Note that the overall computational effort spent on the atomistic simulations was comparable to that for the CG ones.

**Fig 3:**
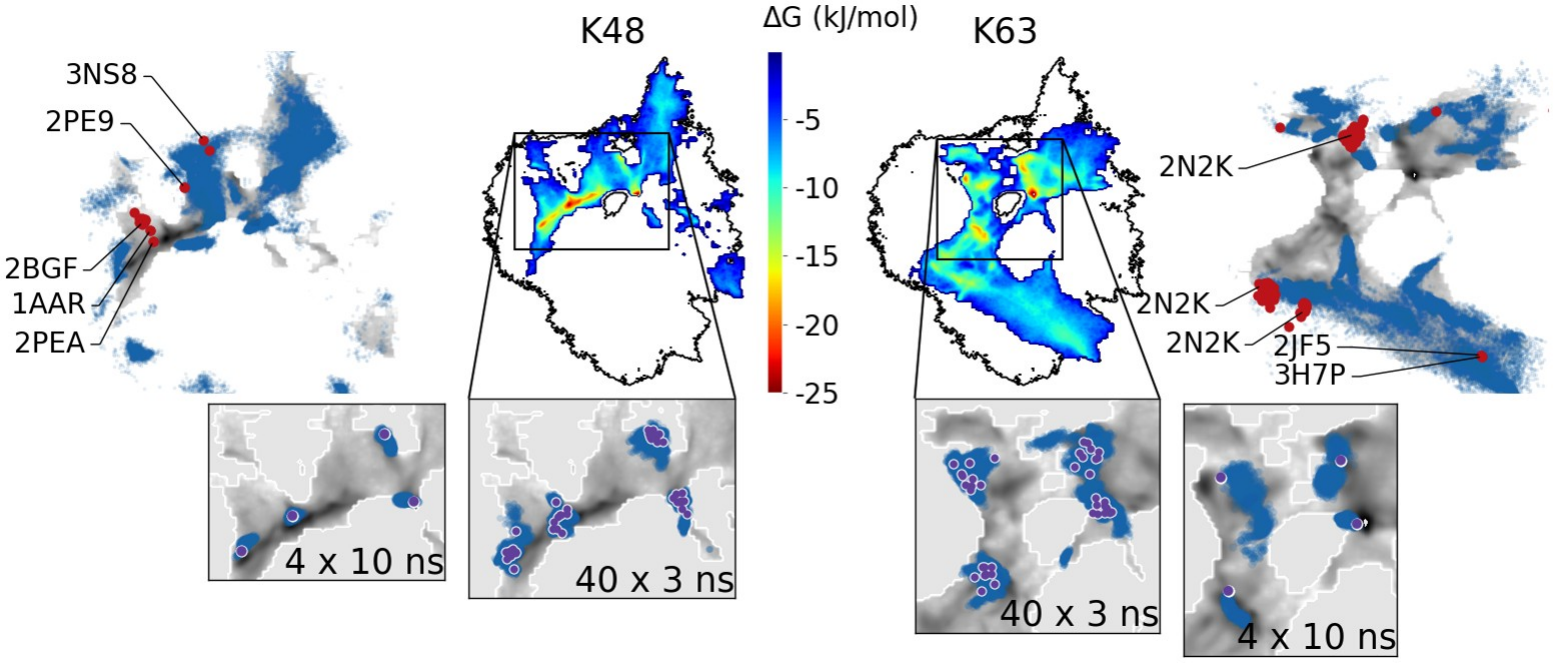
Comparison of atomistic and CG simulations. Colored heatmaps in center: CG energy landscapes of K48- and K63-linked diUb (as in Fig 2). Outer panels: CG data in gray scale with data from atomistic simulations from open initial conformations superimposed as blue dots. Red points: experimental PDB structures. Bottom insets: Zoom with data from atomistic simulations started from back-mapped CG structures as blue dots (violet points: initial structures).

All atomistic simulations were projected into the respective CG free energy landscapes (blue dots in outer panels in Fig 3 and S5 Fig). Naturally, the timescale limitations and the local trapping in metastable states hinders the atomistic sampling which does not completely cover the entire landscape found in the CG simulations. Nevertheless, the overall agreement between the atomistic and the CG sampling is remarkable. The general outline of the areas covered by the free atomistic and CG simulations is very similar. Often the free atomistic simulations get arrested in metastable states before they reach the minima found by the CG models. Validation of these stable structures was therefore done via backmapping. Insets in Fig 3 show that atomistic simulations for K48- and K63-linked diUb, which had been started from backmapped CG structures, do not leave the low-free energy areas of the CG model. In some cases, even the shape of the CG basins is reproduced. A similar agreement is found for the other linkage types (S5 Fig). Further validation of the simulated conformational space is provided by comparison with experimental diUb structures. For K48-linked diUb, CG simulations found a low energy basin (see also S4 Fig) which is very close to the X-Ray structure (PDB-ID: 1AAR) and two NMR structures (PDB-ID: 2BGF, 2PEA). Other experimental structures of K48- and K63-linked diUb (determined under different experimental conditions) lie in regions that had been visited by the CG model, albeit at the border of the simulated landscapes.

### Interpretation of linkage-dependent properties

In the following, we present a more detailed analysis of the conformational space visited by the diUb, with the aim to better understand the differences and similarities between the linkages, identify linkage-dependent surface properties of the chains, and relate them to experimental data.

#### Comparison of projections

**Fig 4:**
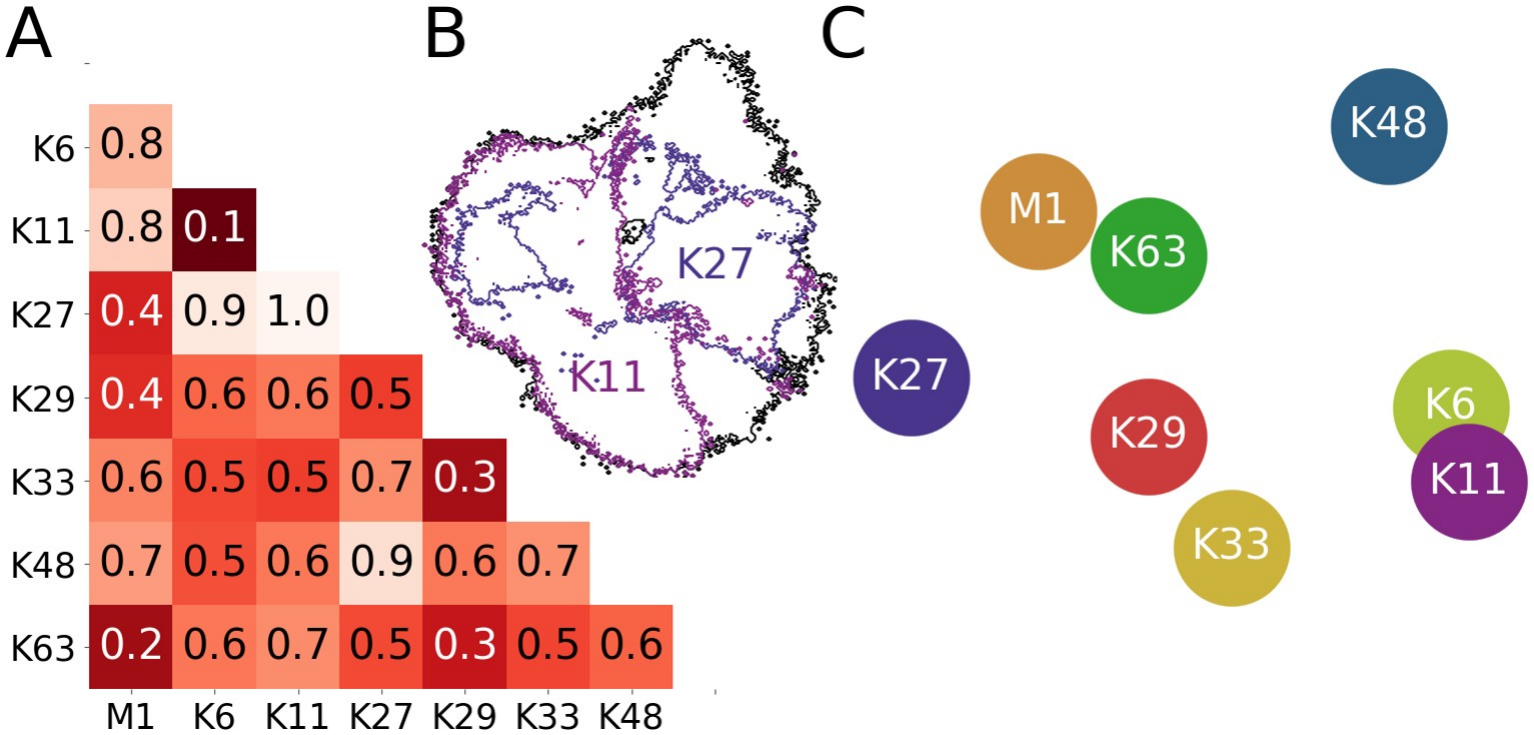
Comparison of 2D projections. (A) Normalized earth mover distances (EMDs) between all projections in Fig 2 (i.e. all linker types). (B) Comparison of conformational landscapes to illustrate the EMD metric: all CG simulations (black); K11- and K27-linked diUb (pink and violet; EMD of 1.0). (C) 2D arrangement of linkage types based on their pairwise EMDs (i.e. according to (dis)similarity).

As a first step, we aimed for a quantitative comparison of the 2D projections of the conformational landscapes. To this end, we tested several metrics that have been developed to assess the similarity of distributions and found that the so-called Earth Mover Distance (EMD) [33–36], widely used in the computer vision and image retrieval community, was particularly well suited for the 2D landscapes (for a detailed description, see S1 Text). The EMD values between all projections in Fig 2 are shown in Fig 4A. A low EMD value indicates a particularly high degree of conformational similarity between two linkages, as is the case for K6 and K11, or K63 and M1, while, in contrast, the conformational landscapes of K11- and K27-linked diUb are very dissimilar (Fig 4B). The similarity/dissimilarity of all linkage types was visualized by arranging them according to their respective pairwise EMDs (again by employing a multi-dimensional scaling technique, see S1 Text). The result of this analysis (Fig 4C) confirms what had already been proposed upon visual inspection of Fig 2. K27, K48, and the K6/K11 pair are positioned in the outer regions of the graph, i.e. they stand out with conformational landscapes that are quite dissimilar to each other. The remaining linkage types (M1/K63 and K29/K33) are positioned more centrally, i.e. they share certain structural features with other linkages. Interestingly, these similarities/dissimilarities correlate well with recent proteomics studies. It was shown, for example, that K27-linked diUb binds to a set of proteins that differs from that binding to K29- and K33-linked diUb [31]. Similarly, K27-and K48-linked diUb bind to different proteins [37], although results obtained in vitro indicate that they may also share common interaction partners [16].

#### The interdomain interface

**Fig 5:**
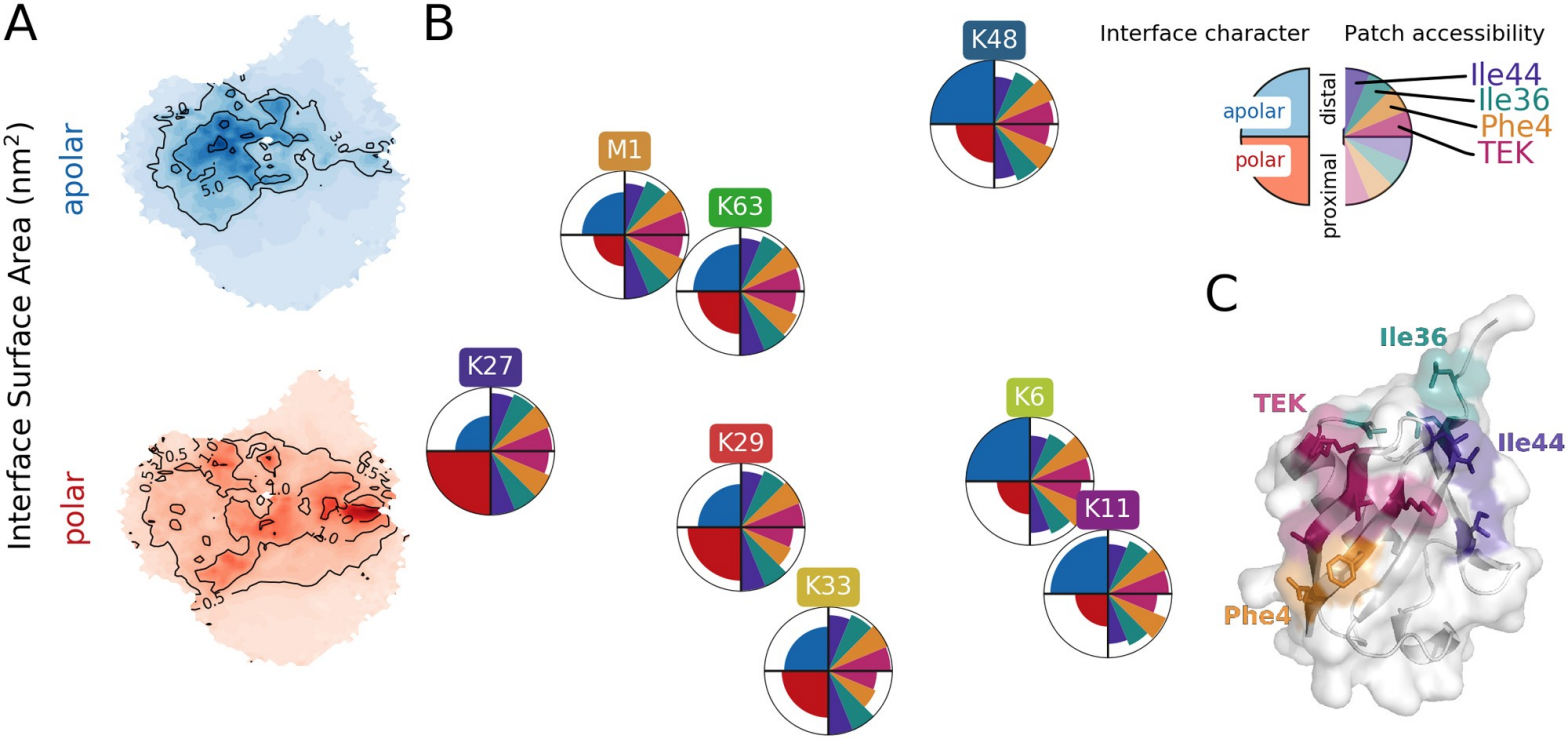
Characteristics of contact interface between Ub subunits from CG simulations. (A) Mean interface surface area between distal and proximal chain on sketch-map projection divided in polar and apolar parts. (B) Circles positioned as in Fig 4C. Left half of circles show interface character, e.g. K27 has the most polar, K6 and K48 the most apolar interface. Right half of circles shows the accessibility of four known interaction patches on the distal and proximal chain. (C) Interaction patches of Ub.

Next, we used the simulation data to obtain a molecular view on the the conformational (dis)similarities found in the mathematical analysis and relate them to linkage type specificity. As shown in Fig 1C, the position on the sketch-map projection can be connected to structural characteristics of the relative position of the two Ub subunits and consequently the part of the surface through which they are interacting. Analysis of the solvent accessible surface area (SASA) can provide insight into the nature of the contacts between the Ub subunits. The interface surface area

$$\begin{array}{c} SA_{\text{interface}}=SASA_{\text{distal}}+SASA_{\text{proximal}}-SASA_{\text{diUb}} \end{array}$$ (1)

can be separated into apolar (a-SA) and polar (p-SA) contributions. Fig 5A shows the diUb landscape which was colored according to the polarity of the SA_interface_ of the respective structures. Areas, where the a-SA or p-SA values are high, e.g. where the interface between the distal and proximal domain is dominated by either hydrophobic or polar contacts, are to some extent separated on the landscape. Comparison with landscapes of certain linkage types (Fig 2) shows that the character of the interface in diUb depends on the linkage position. For example, the landscape sampled by K48-linked diUb coincides with regions in Fig 5A with predominantly apolar contacts between the subunits. In Fig 5B, the interface character is indicated for each linkage type on the left hand side of the circles (which were positioned as in Fig 4C; the size of blue and red areas corresponds to average a-SA and p-SA values; for details, see S1 Text). While K6- and K48-linked diUb form hydrophobic contacts, the inter-domain contacts are mostly polar in K27-linked diUb. The other linkage types lie in between these extreme cases.

In the past, certain regions, so called patches, on the Ub surface were reported, which play an important role in the recognition by proteins harboring ubiquitin-binding domains (UBDs) and which eventually determine the fate of proteins modified by different types of Ub chains [4]. Therefore, it was proposed that the linkage-dependent (in)accessibility of those areas may be a reason for UBD specificity for certain Ub chains. We determined the accessibility of four known Ub patches (Ile44-, Ile36-, Phe4-patch as well as the TEK box [4]) on the simulated diUb structures by comparing their SASA with that in Ub monomers (for details see S1 Text). The right hand side of the circles in Fig 5B shows the obtained patch accessibility estimates for each Ub subunit in all diUbs. Patch accessibility on the distal Ub is comparable for all linkages (except for the distal hydrophobic patches Ile44 and Ile36, which are covered slightly more in K6-, K11- and K48-linked diUb). This limited linkage dependence is not surprising since the distal Ub is connected via its C-terminus, and not a lysine residue. Patch accessibility on the proximal Ub depends more strongly on the linkage position. For K48-linked diUb, the patch coverage is very similar on the distal and proximal subunits, which agrees well with the highly symmetric dimer found in the crystal structure [6]. The Phe4-patch and the TEK-Box are highly covered on the K29- and K33-linked proximal Ub. In M1-, K63- and K27-linked diUb, all patches are covered only moderately. Note that accessibility of individual patches is probably not the only determinant in selective Ub chain recognition, the relative spatial arrangement of several patches on different monomers may also be important. The simulated conformational ensembles now offer the opportunity to search for structures (sub-ensembles) which present the patches in certain arrangements for conformational selection and binding.

#### Residue-wise accessibility

**Fig 6:**
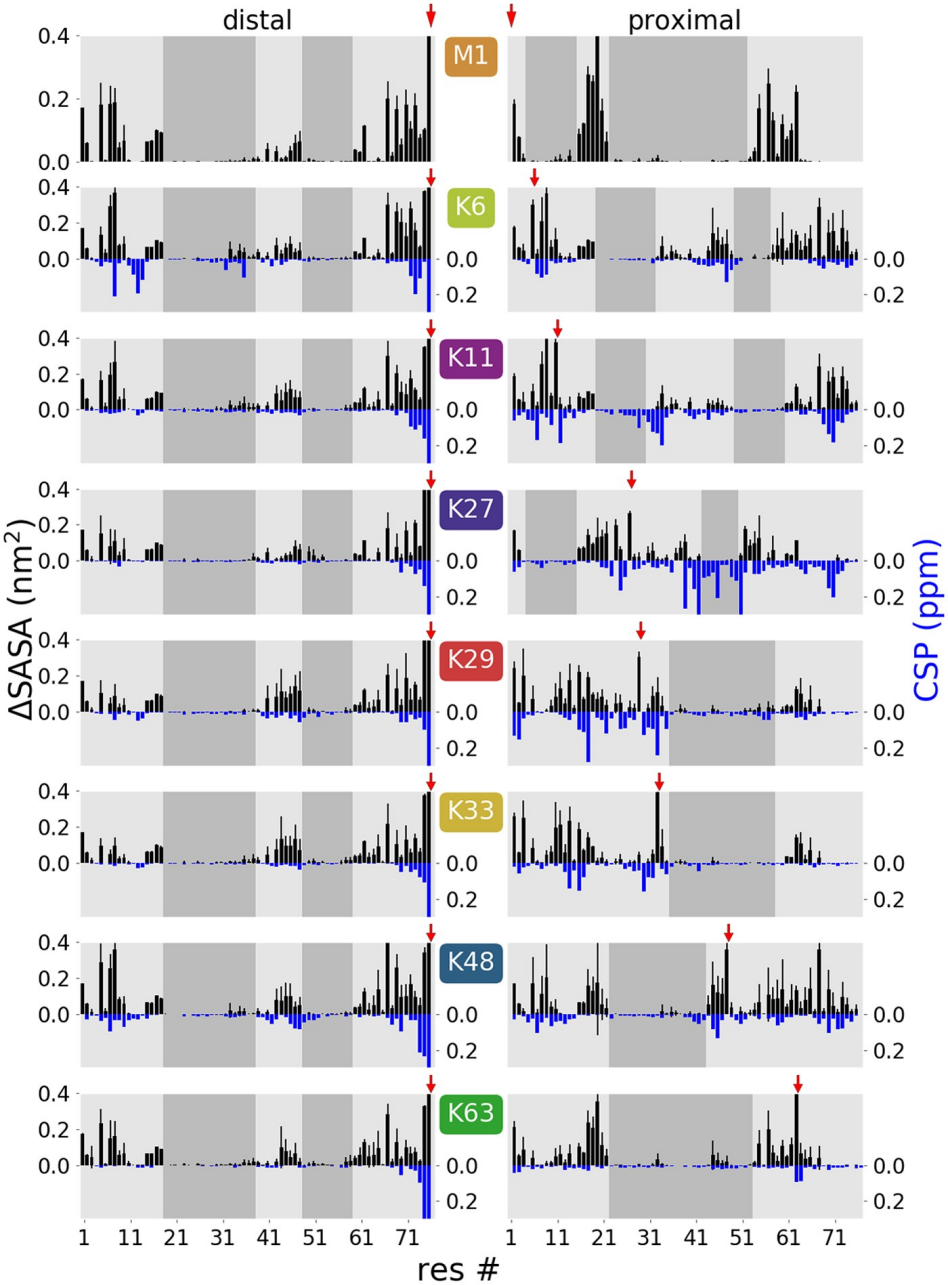
Residue-wise ΔSASA values (black bars, top) compared to NMR data (blue bars, bottom). ΔSASA calculated from CG simulations show the mean loss of accessibility for each residue and therefore the extent of interaction inside the dimer (error bars were estimated from the variance of ΔSASA for the 12 independent simulations for each linkage type). Chemical shift differences (CSP) between the distal (left) or proximal (right) Ub units in the dimer and the monomeric Ub from [15] made available by D. Fushman (no experimental data is available for M1-linked dimers). Shaded areas show regions with low ΔSASA values indicating potential interaction faces for diUb recognition. Red arrows indicate linkage position. Note that high CSP values can in part also originate from chemical modification of the lysine side chain on the proximal subunit and the C-terminus on the distal subunit [15].

Quantitated differences in chemical shifts (CSP) from NMR experiments have been interpreted as an indicator how much a certain residue in one Ub subunit is affected by the proximity of the second Ub in the dimer [15]. For comparison with these data, we calculated from the MD simulations the mean loss of SASA for each residue in one Ub subunit due to the presence of the second Ub (ΔSASA), as a measure which residues of diUb are involved in inter-domain contacts. In Fig 6 the residue-wise ΔSASA values from the CG simulations are presented alongside the CSP values from Castaneda et al. [15], displaying a remarkable correlation for all differently-linked diUbs (though slightly less for K27)—in spite of the methodological differences, by which these data sets were procured. Shaded areas in Fig 6 indicate regions were ΔSASA is low, i.e. which are accessible to solvent (or potential interaction partners). As already seen for the patches (Fig 5B), the change in solvent accessibility of the distal Ub is similar for all linkage types. This is different for the proximal Ub, where the surface accessibility is significantly affected by the linkage type. The high correlation with the experimental CSP data gives further credibility to the simulation ensemble.

## Discussion

By the use of dual-scale MD simulations and a detailed mathematical analysis of the thus obtained conformational ensembles, we obtained insights into the properties of differently linked Ub dimers in solution.

Residue-wise minimum distances turned out to be suitable CVs to represent the conformational space of diUb, in particular with a sketch-map projection into a 2D free energy landscape. We showed that this allows an intuitive examination of the conformational space, as well as qualitative and quantitative assessment of the (dis)similarities of different linkage types. In the present case, we were able to validate data that were obtained from a CG force field with atomistic simulations and compare all native diUb types. This newly developed approach for diUb should be more generally applicable to other problems where domains perform complex movements relative to each other.

For diUb, we found that the character of inter-domain contacts depends strongly on the linkage position. Thus, the surface of Ub, which is accessible for contacts with interaction partners, is altered by ubiquitylation, particularly on the proximal monomer. However, some diUb show very similar behaviour, e.g. K6 and K11 or K29 and K33, which is in agreement with experimental results and confirms the redundant character of the ubiquitin code [15]. Coverage of distal residues is comparable for all linkage types. We therefore conclude that the most distal Ub in a Ub chain makes the least contribution to specificity. Hence, the proximal Ub, which is ubiquitylated itself, holds the major information about the actual function of the respective chain type. This provides a hint why sometimes a certain minimum Ub chain length is required for recognition by UBDs [38]. It may also indicate that deubiquitylating enzymes, which perform distal trimming of Ub chains [39], have to bind to at least two of the very last subunits of a chain to obtain linkage type specificity. In the future, it will be highly interesting to study the behavior of Ub moieties, which are in the interior of a longer chain and consequently should display a mixture of unspecific distal and specific proximal properties. This will extend our knowledge about relevant patterns underlying the Ub code. Work provided here opens up a whole realm of possible applications to questions related to protein-protein interactions inside as well as outside of the Ub signaling system.

## Materials and methods

### MD simulations

All simulations were performed with the GROMACS simulation package v5 [40]. Temperature and pressure were kept at 300 K and 1 bar using the velocity rescaling thermostat and the Parrinello-Rahman barostat, respectively. The Verlet cut-off scheme was applied. The LINKS algorithm was used to constrain all bonds. The default md (leap-frog) integrator was used.

All open initial conformations of diUb were constructed from two Ub units (PDB-ID: 1UBQ) by placing the Ub moieties next to each other so that the C-terminal distal carboxyl group and the proximal lysine side chain were closer than 0.3 nm. For each linkage type, a second conformation was generated. For this, the relative orientation between the distal and proximal Ub was altered. For all simulations, diUb was placed in a 10×10×10 nm dodecahedron box to avoid interactions between periodic copies. All structures were relaxed by energy minimization before and after solvation. Solvated systems were equilibrated in three short runs of 200 ps: (1) under constant temperature (NVT) with a position restrained backbone; (2) under constant temperature and pressure (NPT) with a position restrained backbone; (3) NPT without any position restrains.

### Atomistic simulations

For atomistic MD simulations, the GROMOS96 54a7 force field [41] with the SPC/E water model was used. The integration time step was 2 fs with a cut-off for short range van der Waals interactions of 1.4 nm. Electrostatics were treated with the Particle Mesh Ewald scheme with a 1.4 nm cut-off. Coarse grained diUb structures, which were used for atomistic simulations, were back-mapped with BACKWARD [32].

The force field had to be complemented to enable the simulation of covalently linked dimers via an isopeptide bond. The respective parameters were chosen in analogy to the regular peptide bond of the force field.

### Coarse grained simulations

The MARTINI force field v2.2 [42, 43] was used as basis for all CG simulations. The MARTINI non polarizable coarse grained water was used as solvent. A 10 fs time step could be used due to the soft elastic network potentials. The cut-off distance for short range van der Waals interactions was set to 1.1 nm and electrostatics were treated by the reaction field method with a cut-off distance of 1.1 nm and a dielectric constant of 15.

For nonbonded interactions a modified MARTINI parameter set where all protein-water interactions are increased by 0.35 $\frac{\text{kJ}}{\text{mol}}$ was used (kindly provided by D. H. de Jong, University of Muenster, personal communication). Structure and topology input files for CG simulations were created with the *martinize* script, v2.4 available at the MARTINI project website. For construction of topologies of diUb, this script was modified and functionality for a formation of an isopeptide bond was added. All coarse grained simulations were performed using the ELNEDIN force field [44] for bonded interactions. The MARTINI ff was adapted to describe the structural and dynamic properties of diUb as accurate as possible. This was achieved in three steps. Firstly, by parametrization of an isopeptide linker, which is not available in MARTINI. Secondly, by determination of a favorable secondary structure in solution for assignment of the strength of non-bonded backbone interactions. For further details please see S1 Text. Finally, a supportive network was derived to reproduce the intrinsic dynamic properties of Ub correctly. The Iteratively-refined Distance-based Elastic Network (IDEN) method [45] was used to obtain a supportive network. This approach requires an ensemble of reference conformations which was composed from atomistically simulated structures and already used for secondary structure determination. Best results were achieved using a maximum bond distance of 1.0 nm and an initial force constant of 800 $\frac{\text{kJ}}{\text{mol}}$. Pseudobonds were excluded by a variance threshold of 0.015 nm and explicitly included by a covariance threshold of 0.7 nm. Refinement against distance variance differences was achieved using 50 ns long reference simulations with a scaling factor of 4000 over 30 iteration steps. Pseudobonds with a final force constant of 1 $\frac{\text{kJ}}{\text{mol}}$ or lower were removed from the topology to prevent cut-off errors and thus terminations in subsequent simulations.

### Sketch-map

Sketch-map v3.0 was used. RMD values were computed every 100 ps and 10 ps from CG and atomistic simulations, respectively. Based on the high-dimensional distance distribution of CG data, the sigmoid function parameters *σ* = 5.9, *A* = 12, *B* = 4, *a* = 2, *b* = 4 were chosen. Landmarks (N = 2000) were selected from CG simulations only (S1 Fig). This selection was done randomly in combination with the minmax option with *γ* = 0.1. For further details please see the Results section and S1 Text.

## Supporting information

S1 Text Supplementary information. This file contains further details about the coarse grained simulation setup and data analysis which were performed to produce the figures.
(PDF)

S1 Fig Sketch-map landmarks. Conformations (N = 2000) were selected from CG simulations as landmarks for Sketch-map (red points), after 10 steps of optimization. Contour of landscape of all Ub dimers as black line.
(TIF)

S2 Fig Initial conformations of diUb used for CG and atomistic simulations. Open dimers were constructed from two monomers and positioned in a way that an iso-peptide bond can be formed.
(TIF)

S3 Fig Center of geometry distance of all CG simulations. Distance between Ub moieties inside a dimer as a function of time, separated by linkage type. Tendency to form stable aggregates is linkage dependent.
(TIF)

S4 Fig Structures obtained from low energy areas of CG simulations. CG structures were back-mapped as described in SI text to obtain an atomistic representation. The distal Ub subunit is always at the bottom. Secondary structure motives are colored in yellow (*β*-sheet) and purple (*α*-helix). Hydrophobic patch is shown as blue spheres on both subunits.
(TIF)

S5 Fig Comparison of atomistic and CG simulations. Free-energy landscapes of diUb as colored heatmaps in the middle. Same heatmaps are shown in gray scale on the outer part of the figure. Data from atomistic simulations which were started from open conformations are shown as blue points. Zoomed insets with atomistic simulations started from back-mapped CG structures shown as blue points (start of simulations as violet points).
(TIF)
