# Supplementary material for: Towards a molecular basis of ubiquitin signaling: A dual-scale simulation study of ubiquitin dimers
Source: PLoS Comput Biol. 2018 Nov 16;14(11):e1006589. doi: 10.1371/journal.pcbi.1006589 (PMC6268000; doi:10.1371/journal.pcbi.1006589)
Supplement: S1 Text — This file contains further details about the coarse grained simulation setup and data analysis which were performed to produce the figures. (PDF) [file pcbi.1006589.s001.pdf]

Supplementary Information for: Towards a Molecular Basis of Ubiquitin Signaling: A Dual-Scale Simulation Study of Ubiquitin Dimers

Andrej Berg, Oleksandra Kukharenko, Martin Scheffner, Christine Peter

November 5, 2018

Coarse Grained Simulations

Equilibrium bond lengths and angles for the linkage were obtained from previously performed atomistic simulations of Ub dimers (Tab A). The way of uniting atoms was not changed compared to the initial mapping in MARTINI. Hence, the linkage consists of 4 beads. Furthermore, types of the CG beads were changed to represent the properties of an isopepdide bond. Therefore, types were chosen with mostly polar character as they are assigned in MARTINI to back bone beads without secondary structure. All charges of linkage beads were set to zero.

Tab A. Parameters for a coarse grained isopeptide linkage. Equilibrium distances and angles were calculated from distributions in atomistic simulations. Force constants were adapted from usual values in MARTINI.

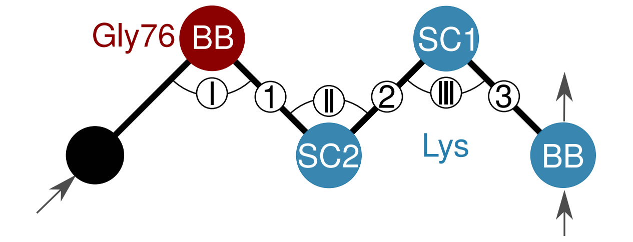

Beads		BB_{76}	SC2	SC1
	Type	P4	P4	C4
Bonds		1	2	3
	$r_0(nm)$	0.302	0.289	0.260
	k $(\frac{\text{kJ}}{\text{mol}})$		150 000	
Angles		I	II	III
	α_0	136	138	145
	k $(\frac{\text{kJ}}{\text{mol}})$		40	

A representative set of Ub conformations from atomistic simulations was used to determine the most common secondary structure and hence assignment of backbone types for non-bonded interactions. Secondary structure dump as provided by GROMACS:

```
1          10         20         30
~EEEEE~TTS ~EEEEE~~TT ~BHHHHHHHH HHHS~~~TTT
40         50         60         70
EEEEETTEE~ ~TT~BTTTTT ~~SS~EEEEE E~~~~~
```

Collective Variables

Residue-wise minimum distances (RMD)

The RMD were calculated with the GROMACS module *mindist*. For two domains A and B with $A = (a_1, a_2, ..., a_n)$ and $B = (b_1, b_2, ..., b_m)$, where a_i and b_j are positions of C_α atoms or backbone beads, computation of pair-wise distances d_{a_i,b_j} gives a $n \times m$ matrix $D_{A,B}$

$$D_{A,B} = \begin{array}{c} \\ y_1 \\ \vdots \\ y_n \end{array} \begin{array}{c} \begin{array}{ccc} x_1 & \dots & x_m \end{array} \\ \begin{pmatrix} d_{a_1,b_1} & \dots & d_{a_1,b_m} \\ \vdots & \vdots & \vdots \\ d_{a_n,b_1} & \dots & d_{a_n,b_m} \end{pmatrix} \end{array}$$

with x_j representing the columns and y_i the rows of the matrix. The RMD results from row- and column-wise minima in a $n + m$ vector

$$RMD_{A,B} = (min(y_1), ..., min(y_n), min(x_1), ..., min(x_m))$$

The number of CVs and hence dimensionality of data is reduced drastically (from $n \times m$ to $n + m$). A full set of pairwise distances, in fact, would be able to describe the relative position of selected atoms with almost no information loss. However, for systems, as in the present case, where large sets of atoms (domains) perform correlated motions, pairwise distances will be highly correlated as well. Compared to an $n \times m$ matrix of all pairwise distances between n atoms in domain A and m atoms in domain B, the dimensionality is reduced to an $n + m$ vector. RMD still contains information on how the Ub moieties are oriented relative to each other and if they are in contact at all. This data is still correlated to some degree but this is handled by sketch-map.

Sketch-map

Sketch-map [1, 2] was shown to be able to project a highly nonlinear conformational space by iteratively minimizing the nonlinear fit function

$$\chi^2 = \sum_{i \neq j} [s(R_{ij}, A, B) - s(r_{ij}, a, b)]^2 \tag{1}$$

and by focusing only on the intermediate distances between points in low and high dimensional spaces applying the sigmoid function

$$s(r_{ij}, a, b) = 1 - (1 + (2^{a/b} - 1)(r_{ij}/\sigma)^a)^{-b/a}, \tag{2}$$

where R_{ij}, r_{ij} are dissimilarities (e.g. Euclidean distances) between points in high- and low-dimensions, respectively. The parameters σ, A, B, a, b are chosen by analyzing the distance distribution between high-dimensional points.

Free Energy Landscapes

Free energy landscapes of CG simulations were calculated from 2D distributions (obtained with Sketch-map) by binning with a grid size of 0.3×0.3 (sketch-map 2D distance) and subsequent Boltzmann inversion

$$\Delta G_{bin} = -k_B \cdot T \cdot N_A \cdot \log(p_{bin})$$

where k_B is the Boltzmann constant, T is 300 K, N_A is the Avogadro constant and p_{bin} is the binned probability distribution. To obtain smoother landscapes a gaussian filter with a radius of 0.6 was applied.

Tab B. Surface area in nm^2 of interface between distal and proximal chain. Values were used to show the interface character in Fig. 5B, left half of circles.

$\mathrm{SA}_{\mathrm{interface}}$	m1	k6	k11	k27	k29	k33	k48	k63
apolar	4.82	7.26	6.48	3.99	4.85	4.98	7.24	5.31
polar	0.78	0.82	0.81	1.60	1.32	1.16	0.96	1.06

Earth Mover Distance

For comparison of 2D distributions, we needed to choose an appropriate measure that is metric (satisfying the three requirements of a metric, i.e. non-negativity, symmetry, and triangular inequality) and desirably not sensitive to the correspondence between bins index and size. The Earth Mover Distance (EMD) [3–6] widely used in the computer vision and image retrieval community satisfies all those requirements.

EMD, also known as the Mallows or Wasserstein distance in statistical literature, is a solution to the transportation problem, where one minimizes costs that must be paid to transform one distribution into the other. One can imagine the distributions as piles of sand, where each grain of sand is a projection of an observed structure. To quantify the difference between two distributions, we measure how far the grains of sand have to be moved so that the two distributions coincide exactly. EMD is the minimal total ground distance traveled weighted by the amount of sand moved. For the formal definition, see e.g. [3–6]. The disadvantage of EMD is the computational complexity $O(n^3 \log n)$, where n is a number of bins. Here we applied the fast approximations, proposed in [4, 5], which is an order of magnitude faster than the original algorithm.

Due to the computational cost, distributions for comparison with the EMD algorithm were computed with a bin size of 1.0×1.0 (sketch-map 2D distance) using the implementation of *emd* in python[1].

Positioning in 2D based on EMD

To give an illustration to the relative positions of the projections we optimally positioned them in 2D space using metric multi-dimensional scaling[2] and normalized (between 0 and 1) EMDs.

Surface Area

Solvent accessible surface area (SASA) was calculated using the double cube lattice method [7], as is implemented in the GROMOS analysis package *sasa* with a probe size of 0.14 nm every 100 ps. The interface character was determined from the interface surface

$$SA_{\mathrm{interface}} = SASA_{\mathrm{distal}} + SASA_{\mathrm{proximal}} - SASA_{\mathrm{diUb}} \tag{3}$$

which was divided in polar and apolar regions based on the charge of the bead: Atoms/beads which have a partial charge between -0.2 and 0.2 e were considered apolar, consequently the rest was considered polar (Tab B). Patch accessibility was calculated as the relative surface area of a patch inside the dimer compared to its mean area in a reference simulation of mono Ub (Tab C).

Other Software

Structures were visualized with *pymol* v1.7.2.1. Figures were created with the python package *matplotlib* v2.1.

[1] https://github.com/wmayner/pyemd
[2] We used python implementation from the package sklearn.manifold.MDS

Tab C. Mean SASA of patches on Ub in nm^2**.** Comparison between area obtained from mono Ub reference simulation and patch area calculated from diUb simulations.

	Ile44	Ile36	Phe4	TEK	Ile44	Ile36	Phe4	TEK
mono Ub	2.16	2.31	2.52	5.03	2.16	2.31	2.52	5.03
diUb	distal				proximal			
M1	1.73	2.08	2.58	4.77	2.16	2.33	2.50	4.54
K6	1.53	1.83	2.55	4.73	1.74	2.03	2.50	4.01
K11	1.67	2.05	2.55	4.75	1.68	2.00	2.46	3.84
K27	1.94	2.21	2.56	4.80	2.13	2.29	2.57	4.55
K29	1.88	2.19	2.55	4.86	2.13	2.31	2.11	4.07
K33	1.87	2.18	2.54	4.85	2.13	2.32	1.99	3.78
K48	1.59	2.01	2.53	4.62	1.84	2.20	2.54	4.30
K63	1.79	2.15	2.53	4.70	2.15	2.33	2.39	4.35

References

1. Ceriotti M, Tribello GA, Parrinello M. Simplifying the Representation of Complex Free-energy Landscapes Using Sketch-map. PNAS. 2011;108(32):13023–13028. doi:10.1073/pnas.1108486108.

2. Ceriotti M, Tribello GA, Parrinello M. Demonstrating the Transferability and the Descriptive Power of Sketch-Map. J Chem Theory Comput. 2013;9(3):1521–1532. doi:10.1021/ct3010563.

3. Rubner Y, Tomasi C, Guibas LJ. The Earth Mover's Distance as a Metric for Image Retrieval. Int J Comput Vision. 2000;40(2):99–121. doi:10.1023/A:1026543900054.

4. Pele O, Werman M. A linear time histogram metric for improved sift matching. In: Computer Vision–ECCV 2008. Springer; 2008. p. 495–508.

5. Pele O, Werman M. Fast and robust earth mover's distances. In: 2009 IEEE 12th International Conference on Computer Vision; 2009. p. 460–467.

6. Applegate D, Dasu T, Krishnan S, Urbanek S. Unsupervised Clustering of Multidimensional Distributions Using Earth Mover Distance. In: Proceedings of the 17th ACM SIGKDD. ACM; 2011. p. 636–644. Available from: http://doi.acm.org/10.1145/2020408.2020508.

7. Eisenhaber F, Lijnzaad P, Argos P, Sander C, Scharf M. The Double Cubic Lattice Method: Efficient Approaches to Numerical Integration of Surface Area and Volume and to Dot Surface Contouring of Molecular Assemblies. J Comput Chem. 1995;16(3):273–284. doi:10.1002/jcc.540160303.
