# Supplementary figures and images for: Towards a molecular basis of ubiquitin signaling: A dual-scale simulation study of ubiquitin dimers
Source: PLoS Comput Biol. 2018 Nov 16;14(11):e1006589. doi: 10.1371/journal.pcbi.1006589 (PMC6268000; doi:10.1371/journal.pcbi.1006589)

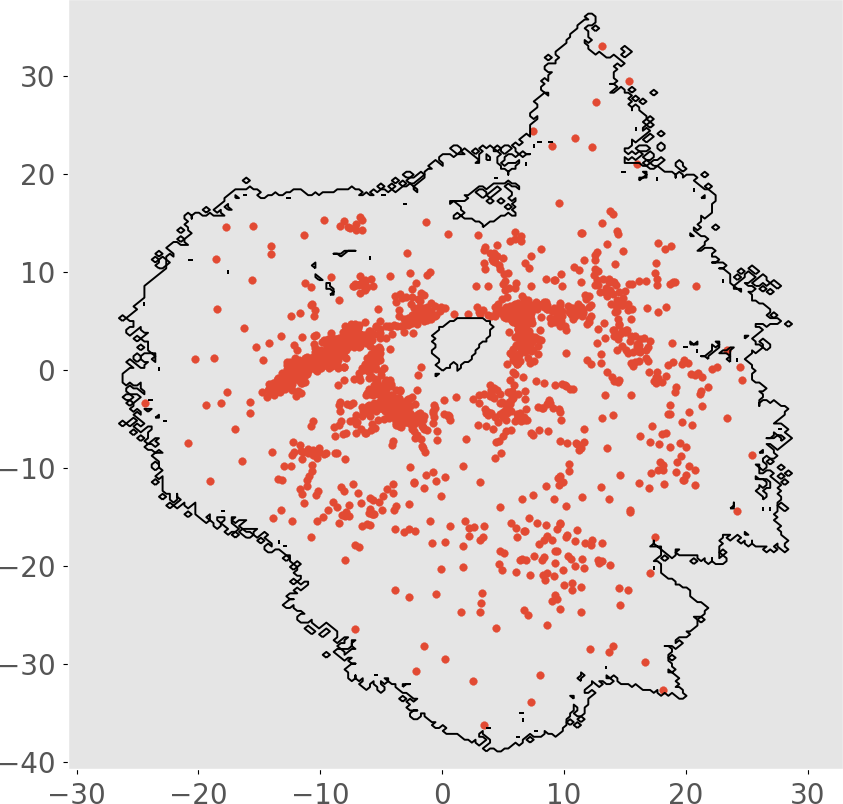

Supplement: S1 Fig — Conformations (N = 2000) were selected from CG simulations as landmarks for Sketch-map (red points), after 10 steps of optimization. Contour of landscape of all Ub dimers as black line. (TIF) [file pcbi.1006589.s002.tif]

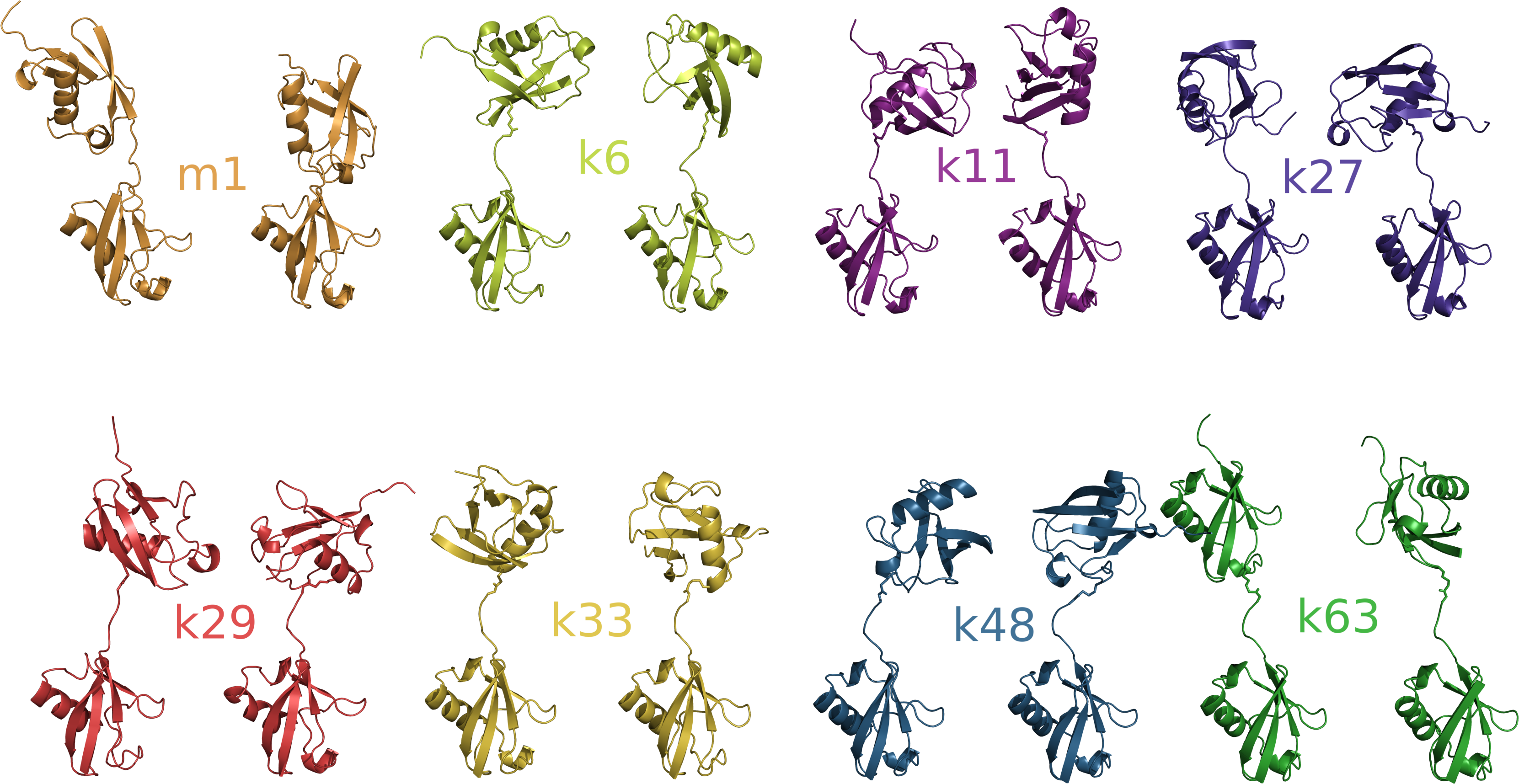

Supplement: S2 Fig — Open dimers were constructed from two monomers and positioned in a way that an iso-peptide bond can be formed. (TIF) [file pcbi.1006589.s003.tif]

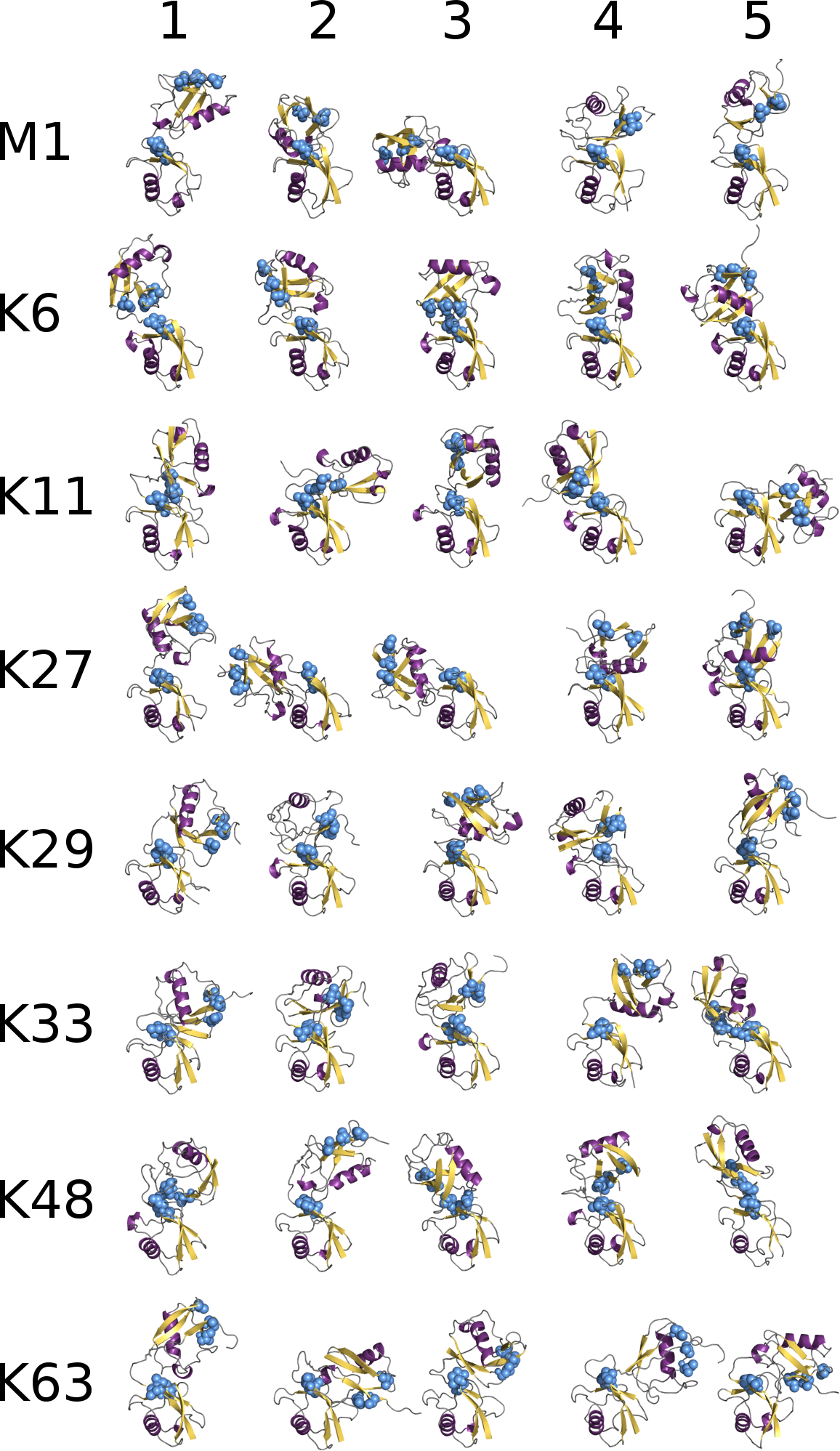

Supplement: S4 Fig — CG structures were back-mapped as described in SI text to obtain an atomistic representation. The distal Ub subunit is always at the bottom. Secondary structure motives are colored in yellow (β-sheet) and purple (α-helix). Hydrophobic patch is shown as blue spheres on both subunits. (TIF) [file pcbi.1006589.s005.tif]

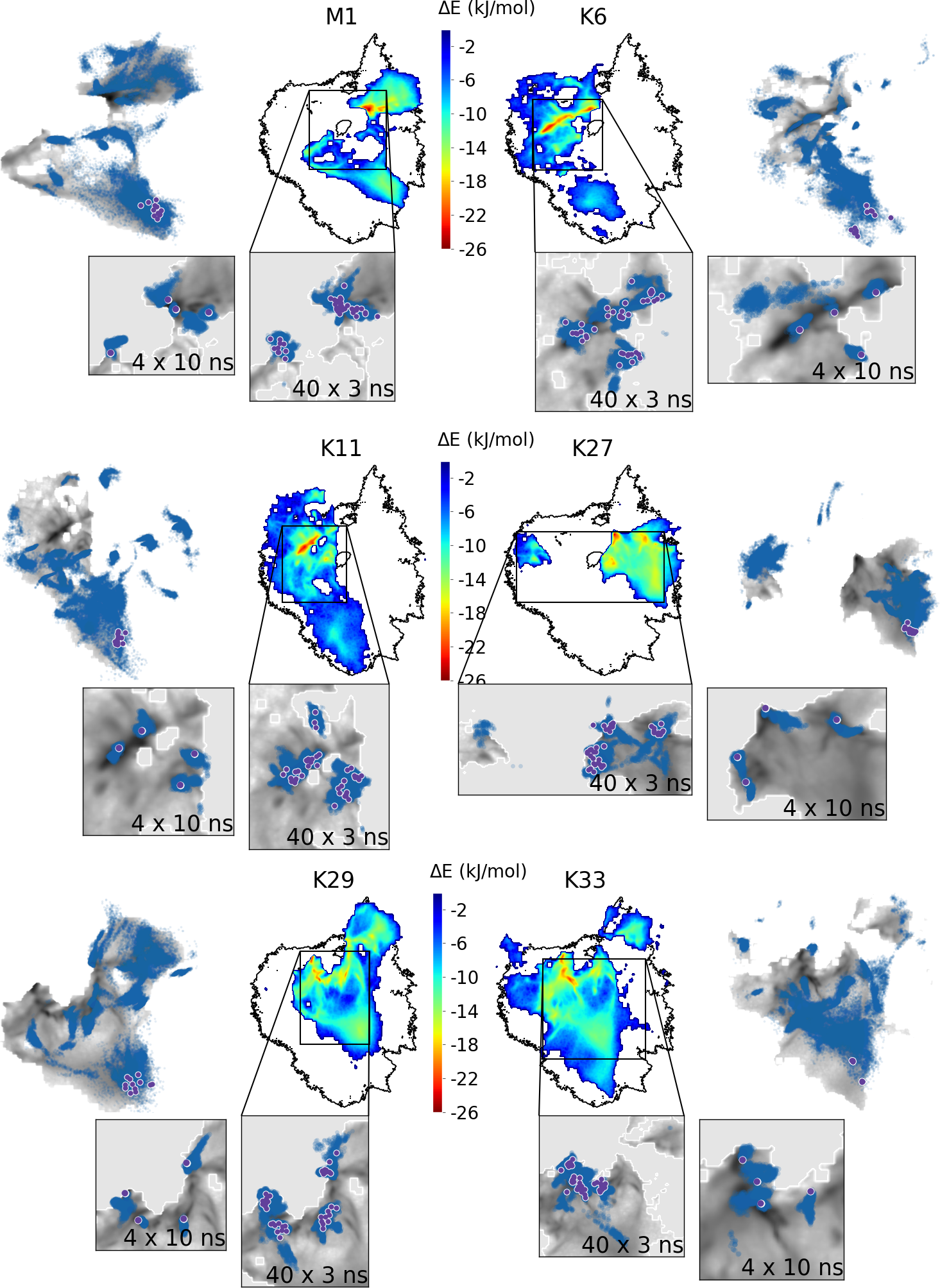

Supplement: S5 Fig — Free-energy landscapes of diUb as colored heatmaps in the middle. Same heatmaps are shown in gray scale on the outer part of the figure. Data from atomistic simulations which were started from open conformations are shown as blue points. Zoomed insets with atomistic simulations started from back-mapped CG structures shown as blue points (start of simulations as violet points). (TIF) [file pcbi.1006589.s006.tif]
